# The prognostic value of nestin expression in newly diagnosed glioblastoma: Report from the Radiation Therapy Oncology Group

**DOI:** 10.1186/1748-717X-3-32

**Published:** 2008-09-25

**Authors:** Prakash Chinnaiyan, Meihua Wang, Amyn M Rojiani, Philip J Tofilon, Arnab Chakravarti, K Kian Ang, Hua-Zhong Zhang, Elizabeth Hammond, Walter Curran, Minesh P Mehta

**Affiliations:** 1 Department of Radiation Oncology, H. Lee Moffitt Cancer Center and Research Institute, Tampa, USA; 2 Department of Statistics, American College of Radiology, Philadelphia, USA; 3 Department of Pathology, H. Lee Moffitt Cancer Center and Research Institute, Tampa, USA; 4 Department of Drug Discovery, H. Lee Moffitt Cancer Center and Research Institute, Tampa, USA; 5 Department of Radiation Oncology, Massachusetts General Hospital/Harvard Medical School, Boston, USA; 6 Department of Radiation Oncology, MD Anderson Cancer Center, Houston, USA; 7 Department of Pathology, LDS Hospital, Salt Lake City, USA; 8 Department of Radiation Oncology Emory University, Atlanta, USA; 9 Department of Human Oncology, University of Wisconsin Hospitals, Madison, USA

## Abstract

**Background:**

Nestin is an intermediate filament protein that has been implicated in early stages of neuronal lineage commitment. Based on the heterogeneous expression of nestin in GBM and its potential to serve as a marker for a dedifferentiated, and perhaps more aggressive phenotype, the Radiation Therapy Oncology Group (RTOG) sought to determine the prognostic value of nestin expression in newly diagnosed GBM patients treated on prior prospective RTOG clinical trials.

**Methods:**

Tissue microarrays were prepared from 156 patients enrolled in these trials. These specimens were stained using a mouse monoclonal antibody specific for nestin and expression was measured by computerized quantitative image analysis using the Ariol SL-50 system. The parameters measured included both staining intensity and the relative area of expression within a specimen. This resulted into 3 categories: low, intermediate, and high nestin expression, which was then correlated with clinical outcome.

**Results:**

A total of 153 of the 156 samples were evaluable for this study. There were no statistically significant differences between pretreatment patient characteristics and nestin expression. There was no statistically significant difference in either overall survival or progression-free survival (PFS) demonstrated, although a trend in decreased PFS was observed with high nestin expression (p = 0.06).

**Conclusion:**

Although the correlation of nestin expression and histologic grade in glioma is of considerable interest, the presented data does not support its prognostic value in newly diagnosed GBM. Further studies evaluating nestin expression may be more informative when studied in lower grade glioma, in the context of markers more specific to tumor stem cells, and using more recent specimens from patients treated with temozolomide in conjunction with radiation.

## Background

Nestin is an intermediate filament protein that was initially identified during studies involving cellular organization of the developing rat nervous system [1]. It was described as the antigen to the monoclonal antibody Rat-401 that specifically identified transient radial glial cells, which guided neuronal migration. It was later cloned in humans and its gene product defined a distinct sixth class of intermediate filament proteins [2]. Nestin expression has been demonstrated in neuroepithelial stem cells and progenitor cells in the human brain and implicated in early stages of lineage commitment. Further, as these precursor cells differentiate along their respective neural or glial cell types, nestin expression has been shown to be down regulated [2-4].

Although not a definitive neural stem cell marker [5], nestin is expressed in the minor-population of tumor stem cells derived from brain tumors that have recently been shown to contribute towards tumorigenicity [5] and therapeutic resistance [6] in glioblastoma (GBM). Although very little is known about the function of nestin, it has been implicated in the distribution and organization of critical cellular factors regulating cell proliferation, survival, and differentiation [7-10]. In addition, nestin has been shown to act as a scaffold protein that regulates the activities of kinases, therefore a potential organizer of survival-determining signaling molecules [9]. However, whether nestin expression is merely a marker of a dedifferentiated state or has a specific biologic function in GBM, remains unclear.

Dalhstrand et al [11] and Tohymama et al [12] performed initial investigations that identified diffuse nestin expression in glioma. Interestingly, these early studies identified higher levels of nestin expression in GBM than in lower grade gliomas [11], supporting its potential application as a marker for dedifferentiation in glioma. Despite the general increased expression of nestin in GBM, staining patterns are heterogeneous, with a proportion of GBM samples demonstrating little to no expression of nestin [11,13-17]. The clinical relevance of these varying expression patterns of nestin in GBM has not been defined. Based on the heterogeneous expression of nestin in GBM and its potential to serve as a marker for a dedifferentiated, and perhaps more aggressive phenotype, the RTOG sought to determine the prognostic value of nestin expression in newly diagnosed GBM patients treated on prior prospective RTOG clinical trials.

## Methods

### Study population

Table 1 lists the specific RTOG trials represented in this correlative study (RTOG 7401, 7918, 8302, 8409, 9006, 9305, 9602, 9806). Patients were generally treated by surgical resection, followed by external beam radiotherapy with or without chemotherapy. The specific chemotherapeutic and other experimental interventions in these trials did not appear to influence survival times. Table 2 presents the relevant demographic data of the 153 patients with GBM treated on previous RTOG clinical trials who had tissue blocks adequate to generate tissue microarrays (TMAs) for the present analysis. TMAs were prepared and evaluated as previously described [18].

**Table 1:** RTOG studies included in analysis

Study	Phase	Description	N = 153
7401	III	WBRT+(BCNU vs. MeCCNU+DTIC)	38 (25%)
7918	III	WBRT+(BCNU vs. Misonidazole radiosensitizer & BCNU)	9 (6%)
8302	III	Hyperfractionated and Accelerated RT + BCNU	30 (20%)
8409	I/II	WBRT + AZQ (NSC-182986)	1 (1%)
9006	III	BCNU + (Hyperfractionated RT vs. RT)	32 (21%)
9305	III	+/-SRS followed by RT+BCNU	3 (2%)
9602	II	RT + Taxol	13 (8%)
9806	II	RT + Thalidomide	27 (18%)

**Table 2:** Patient characteristics by study

Characteristics	7401 (n = 38)	7918 (n = 9)	8302 (n = 30)	8409 (n = 1)	9006 (n = 32)	9305 (n = 3)	9602 (n = 13)	9806 (n = 27)	Total (N = 153)
Gender									
Male	27 (71%)	4 (44%)	22 (73%)	1 (100%)	20 (63%)	1 (33%)	5 (38%)	18 (67%)	98 (64%)
Female	11 (29%)	5 (56%)	8 (27%)	0	12 (38%)	2 (67%)	8 (62%)	9 (33%)	55 (36%)

Race									
White	35 (92%)	8 (89%)	28 (93%)	1 (100%)	30 (94%)	3 (100%)	13(100%)	26 (96%)	144 (94%)
Hispanic	2 (5%)	0	0	0	0	0	0	1 (4%)	3 (2%)
Black	1 (3%)	1 (11%)	1 (3%)	0	2 (6%)	0	0	0	5 (3%)
Other	0	0	1 (3%)	0	0	0	0	0	1 (1%)

Neuro. Function (Symptoms)									
None/Minor	16 (42%)	3 (33%)	13 (43%)	0	23 (72%)	0	7 (54%)	19 (70%)	84 (54%)
Moderate	13 (34%)	5 (56%)	16 (53%)	1 (100%)	9 (28%)	3 (100%)	6 (46%)	8 (30%)	59 (38%)
Major/Severe	8 (21%)	1 (11%)	1 (3%)	0	0	0	0	0	11 (7%)
Missing	1 (3%)	0	0	0	0	0	0	0	1 (1%)

KPS									
≤ 60	11 (29%)	1 (11%)	2 (7%)	0	1 (3%)	0	0	2 (7%)	17 (11%)
70–80	13 (34%)	6 (67%)	19 (63%)	1 (100%)	12 (38%)	0	6 (46%)	10 (37%)	67 (44%)
90–100	14 (37%)	2 (22%)	9 (30%)	0	19 (59%)	3 (100%)	7 (54%)	15 (56%)	69 (45%)

Prior Surgery									
Biopsy	5 (13%)	0	1 (3%)	0	1 (3%)	0	0	3 (11%)	10 (7%)
Part. Resect.	21 (55%)	5 (56%)	22 (73%)	1 (100%)	15 (47%)	1 (33%)	10 (77%)	15 (56%)	90 (59%)
Tot. Resect.	11 (29%)	4 (44%)	7 (23%)	0	16 (50%)	2 (67%)	3 (23%)	8 (30%)	51 (33%)
Unknown	1 (3%)	0	0	0	0	0	0	1 (4%)	2 (1%)

RPA									
III	6 (16%)	1 (11%)	3 (10%)	0	7 (22%)	2 (67%)	4 (31%)	6 (22%)	29 (19%)
IV	15 (39%)	3 (33%)	13 (43%)	1 (100%)	16 (50%)	1 (33%)	3 (23%)	11 (41%)	63 (41%)
V	17 (45%)	5 (56%)	14 (47%)	0	9 (28%)	0	6 (46%)	10 (37%)	61 (40%)

### Nestin immunohistochemical staining

Tissue microarrays were processed using a Ventana Discovery XT automated system (Ventana Medical Systems, Tucson) as per manufacturer's protocol with proprietary reagents. Briefly, slides were deparaffinized on the automated system with EZ Prep solution (Ventana). Heat-induced antigen retrieval method was used in Cell Conditioning solution (CC1, Ventana). The mouse monoclonal antibody that reacts to nestin (ab22035, abcam) was used at a 1:900 concentration in Dako antibody diluent and incubated for 60 min. The Ventana Universal Secondary Antibody was used for 32 min at 37°C. The detection system used was the Ventana DABMap kit and slides were then counterstained with Hematoxylin. Slides were then dehydrated and coverslipped as per normal laboratory protocol.

### Quantification of nestin expression

Slides were bar-coded and blinded for automated slide scan imaging and processing. The Ariol SL-50 (version 3.1.2) from Applied Imaging is an automated slide scanner capable of high-throughput slide analysis designed for accuracy, repeatability and objectivity. The system's built in classifiers include the analysis capability for nuclear, cytoplasmic and membranous event classification with trainable software. It uses an Olympus BX-61 upright microscope to provide high-quality images at 1.25×, 5×, 10×, 20× and 40× objectives.

**Figure 1:**
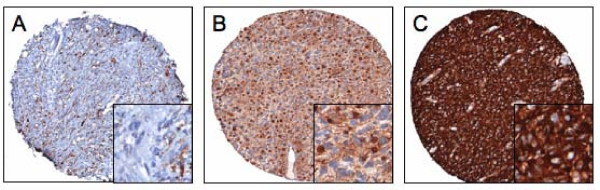
**Varying levels of nestin expression in GBM**. Images are representative of samples categorized as low (A), intermediate (B), and high (C) nestin expression.

Detailed images were processed using the TMA Multistain Imaging Module for the nestin stained brain tissue microarrays slides. The TMAs were processed using the TMA specific imaging assay, TMA Multistain. This allows the software to distinguish positive tumor areas within individual cores of the TMA slide. Both staining intensity and its relative area within a specimen were quantified. Staining intensity was acquired in a continuous gradient and divided into tertiles defined as negative (0), lightly positive (1+), moderately positive (2+) and highly positive (3+) regions. The area occupied by each of these 4 categories was determined, and divided into similar tertiles. A score of 3, 2, 1, and 0 was designated to relative areas ≥ 50%, 33–49%, 1–33%, and 0%, respectively, within the evaluated area of the specimen. This allowed the software to automatically quantitate not only the average intensity of each category, but also the relative area of these stains. The products of the scoring system described above (relative intensity × area) yielded values ranging from 0 to 9, with higher scores reflecting more quantified nestin expression. The highest score of the individual products was used for analysis. Low, intermediate, and high expression was defined as scores ranging from 0–3, 4–6, and 7–9, respectively. Representative samples for low, intermediate, and high expression are shown in Figure 1. All specimens were manually reviewed by a neuropathologist (AMR) to verify overall quality of staining of the tissue microarray and ensure appropriate evaluation of tumor tissue versus necrosis, vessels, and/or other potential aberrances in individual specimens.

### Statistical analysis

This analysis included 153 evaluable patients from 8 RTOG GBM studies. Frequency distributions for the patient characteristics in the three nestin expression levels were compared using χ^2 ^tests of homogeneity. Kaplan-Meier method was used to estimate the OS and the PFS rates and the log-rank test to compare them between the three patient groups. An event for OS is death due to any cause. An event for PFS is recurrence, progression, or death. The Cox proportional hazards model was used to estimate the hazard ratio (HR) associated with each endpoint. A two-sided test was used at a significance level 0.05 for testing.

## Results

**Figure 2:**
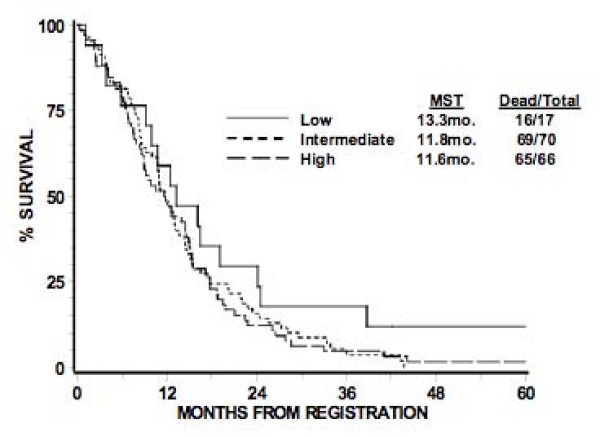
**Kaplan-meier estimates of overall survival according to level of nestin expression**. Nestin expression, stratified as low, intermediate, or high expression, appears to have no statistically significant relationship with overall survival.

**Figure 3:**
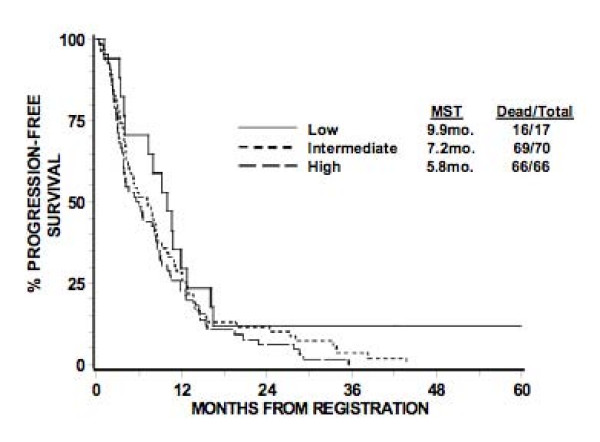
**Kaplan-meier estimates of progression-free survival according to level of nestin expression**. Nestin expression, stratified as low, intermediate, or high expression, appears to have no statistically significant relationship with progression-free survival.

The original RTOG TMA consisted of 156 GBM patients, of which, 153 were evaluable. Of these, the total number of patients that comprised the low, intermediate, and high expression groups were 17, 70, and 66 patients, respectively. The pretreatment characteristics of patients in these three groups appear in Table 3. There were no statistically significant differences seen between the three groups, although there is a trend towards more of the patients with intermediate nestin expression level in RPA III (p = 0.08). When the three groups were compared with regards to OS and PFS based on the log-rank test, no differences were seen at the significance level of 0.05 (Table 4 and 5). Corresponding Kaplan-Meier survival curves are shown in Figures 2, 3. The 12-month survival rates for the patients with low, intermediate, and high nestin expression were 59%, 49%, and 48% respectively. The 12-month PFS rates for the patients with low, intermediate, and high nestin expression were 29%, 27%, and 23% respectively.

**Table 3:** Patient characteristics

Characteristics	Low (n = 17)	Intermediate (n = 70)	High (n = 66)	p-value*
Gender				0.17
Male	13 (76%)	48 (69%)	37 (56%)	
Female	4 (24%)	22 (31%)	29 (44%)	

Race				0.88
White	15 (88%)	66 (94%)	63 (95%)	
Hispanic	1 (6%)	1 (1%)	1 (2%)	
Black	1 (6%)	2 (3%)	2 (3%)	
Other	0	1 (1%)	0	

Neurologcal Function (Symptoms)				0.69
None/Minor	7 (41%)	40 (57%)	37 (56%)	
Moderate	8 (47%)	25 (36%)	25 (38%)	
Major/Severe	2 (12%)	5 (7%)	3 (5%)	
Missing	0	0	1 (1%)	

KPS				0.41
≤ 60	4 (24%)	7 (10%)	6 (9%)	
70–80	8 (47%)	29 (41%)	30 (45%)	
90–100	5 (29%)	34 (49%)	30 (45%)	

Prior Surgery				0.84
Biopsy	0	5 (7%)	5 (8%)	
Partial Resection	11 (65%)	42 (60%)	37 (56%)	
Total Resection	6 (35%)	22 (31%)	23 (35%)	
Unknown	0	1 (1%)	1 (1%)	

RPA				0.08
III	3 (18%)	20 (28%)	6 (9%)	
IV	7 (41%)	25 (36%)	31 (47%)	
V	7 (41%)	25 (36%)	29 (44%)	

Study				
7401	6 (35%)	22 (31%)	10 (15%)	
7918	3 (18%)	4 (6%)	2 (3%)	
8302	4 (23%)	16 (23%)	10 (15%)	
8409	0	1 (1%)	0	
9006	1 (6%)	13 (19%)	18 (27%)	
9305	1 (6%)	1 (1%)	1 (1%)	
9602	2 (12%)	3 (4%)	8 (12%)	
9806	0	10 (14%)	17 (26%)	

* Chi-square Tests

**Table 4:** Overall survival

	Low		Intermediate		High	
Months	% Alive	No. at Risk	% Alive	No. at Risk	% Alive	No. at Risk

0	100%	17	100%	70	100%	66
12	59%	10	49%	34	48%	32
24	29%	5	16%	11	12%	8
36	18%	3	5%	3	5%	3
48	12%	1	0	0	2%	1
60	12%	1	0	0	2%	1

Median Dead/Total	13.3 mo. 16/17		11.8 mo. 69/70		11.6 mo. 65/66	

Pairwise Comparisons using Log-rank test: Low vs. Intermediate (0.18), Low vs. High (0.16), Intermediate vs. High (0.80).

**Table 5:** Progression-free survival

	Low		Intermediate		High	
Months	% Alive	No. at Risk	% Alive	No. at Risk	% Alive	No. at Risk

0	100%	17	100%	70	100%	66
12	29%	5	27%	19	23%	15
24	12%	2	11%	8	6%	4
36	12%	2	4%	2	0	0
48	12%	1	0	0	0	0
60	12%	1	0	0	0	0

Median Dead/Total	9.9 mo. 16/17		7.2 mo. 69/70		5.8 mo. 66/66	

Pairwise Comparisons using Log-rank test: Low vs. Intermediate (0.2), Low vs. High (0.08), Intermediate vs. High (0.31).

Tables 6 and 7 presents the OS and PFS results based on the Cox proportional hazard model. No difference was found in OS with nestin expression level [intermediate vs. low: HR = 1.66 (0.94, 2.93), p = 0.98; high vs. low: HR = 1.47 (0.83, 2.60), p = 0.18], even after adjusting for RPA class [IV vs. III: HR = 1.65 (1.03, 2.66), p = 0.04; V vs. III: HR = 2.58 (1.60, 4.15), p < 0.0001]. The global test for the interaction of nestin expression level and RPA class was not statistically significant (P < 0.001). No difference was found in PFS with nestin expression level [intermediate vs. low: HR = 1.47 (0.84, 2.59), p = 0.18; high vs. low: HR = 1.73 (0.98, 3.06), p = 0.06] without including RPA class, which was not statistically significant.

**Table 6:** Cox proportional hazards model for overall survival

Covariates	Comparison	HR(95% CI)	p-value
Nestin	Low	--	
	Intermediate	1.66 (0.94, 2.93)	0.98
	High	1.47 (0.83, 2.60)	0.18
			
RPA	III	--	
	IV	1.65 (1.03, 2.66)	0.04
	V	2.58 (1.60, 4.15)	< .0001

**Table 7:** Cox proportional hazards model for progression-free survival

Covariates	Comparison	HR(95% CI)	p-value
Nestin	Low	--	
	Intermediate	1.47 (0.84, 2.59)	0.18
	High	1.73 (0.98, 3.06)	0.06

## Discussion

Although initially identified in glioma, nestin expression has since been demonstrated in several other malignancies, including angiosarcoma, gastrointestinal stromal tumors (GIST) [19], hemangioblastomas [20], melanoma [21,22], and basal epithelial breast cancer [23]. Interestingly, in many of these tumors, including glioma, nestin expression has been shown to correlate with advanced grade [11,13-17,19,23,24], supporting its application as a marker for dedifferentiation. As these dedifferentiated or progenitor cells have been implicated in both tumorigenesis [5] and therapeutic resistance [6], we sought to determine if nestin expression level could be used as a clinically relevant prognosticator in GBM.

The presented data evaluates the prognostic impact of nestin expression in GBM. Other investigators have suggested a more definitive correlation of nestin expression with decreased overall survival [14,17], although these studies included all high-grade glioma. With the known correlation of increased nestin expression with higher-grade glioma, coupled with the known prognostic value of tumor grade alone in glioma, it is unclear if nestin expression would retain its prognostic value in these studies if tumor grade was considered independently.

Based on the presented findings, total nestin expression level, as measured immunohistochemically, does not appear to demonstrate a statistically significant difference in OS or PFS in newly diagnosed GBM. However, there are potential limitations to the interpretation of these results. As the tissue microarray used in this study was created retrospectively from all available tissue from the respective trials, one could make the valid argument that this population would be enriched with patients undergoing a complete or partial resection versus biopsy alone, and therefore this cohort may not be an appropriate representation of all GBM. Secondly, the archived tissue spans over 20 years from patients enrolled on a variety of different therapeutic regimens, although clinical outcome did not appear to be altered. And lastly, and perhaps the most important, these findings are only relevant to the pre-temozolomide era. As the standard of care has since shifted, it would be of value to revisit these studies in this context. Along these lines, defining the relationship of nestin expression with the promoter methylation status of MGMT would also be of considerable value [25,26].

In addition, studies focused on nestin expression in low-grade glioma may also have more definitive clinical applications. Our data (not included) as well as others [11,14-17,24], have shown in low-grade glioma, despite a higher proportion of tumors demonstrating low nestin expression, a significant number of these specimens do express nestin highly. With the significantly more heterogeneous clinical outcomes in low-grade glioma, defining the prognostic value of nestin in this population would be of particular interest. For example, in this context, nestin expression may potentially serve as a biologic marker for a high-risk low-grade glioma, which could have a direct clinical application.

## Conclusion

Although the correlation of nestin expression and histologic grade in glioma is of considerable interest, the presented results do not support its influence on prognosis in GBM patients. Nestin appears to define a dedifferentiated state, although is not a definitive neural stem cell marker [5]. Therefore, nestin expression may have a limited role in identifying the specific cancer stem cell populations within a tumor. This is further supported by the diffuse staining of nestin in our specimens, as opposed to cancer stem cells, which purportedly represent only a minor-fraction of the entire brain tumor cell population. Therefore, further studies evaluating nestin expression in GBM may be more informative when studied in the context of markers more specific to tumor stem cells, including CD133 [5]. In addition, future investigations evaluating more recent specimens from patients treated during the temozolomide era in conjunction with MGMT promoter methylation status may have a more direct clinical relevance.

## Competing interests

The authors declare that they have no competing interests.

## Authors' contributions

PC and AMR manually reviewed all histological sections. PC, MW, and PJT were involved in the initial research concept and draft. MW performed all statistical analysis. AC, KKA, HZ, EH, WC, and MPM were involved in creation of TMAs used in this study. All authors read and approved the final manuscript
